# Activation of the inflammatory transcription factor nuclear factor interleukin-6 during inflammatory and psychological stress in the brain

**DOI:** 10.1186/1742-2094-10-140

**Published:** 2013-11-26

**Authors:** Franziska Fuchs, Jelena Damm, Rüdiger Gerstberger, Joachim Roth, Christoph Rummel

**Affiliations:** 1 Department of Veterinary-Physiology and -Biochemistry, Justus-Liebig-University Giessen, Frankfurter Strasse 100, Giessen D-35392, Germany

**Keywords:** Nuclear factor interleukin-6, Lipopolysaccharide, Pituitary, Tumor necrosis factor α, Hypothalamic paraventricular nucleus, Novel environment-stress, STAT3, NFκB, Body temperature, Activity

## Abstract

**Background:**

The transcription factor nuclear factor interleukin 6 (NF-IL6) is known to be activated by various inflammatory stimuli in the brain. Interestingly, we recently detected NF-IL6-activation within the hypothalamus-pituitary-adrenal (HPA)-axis of rats after systemic lipopolysaccharide (LPS)-injection. Thus, the aim of the present study was to investigate whether NF-IL6 is activated during either, inflammatory, or psychological stress in the rat brain.

**Methods:**

Rats were challenged with either the inflammatory stimulus LPS (100 μg/kg, i.p.) or exposed to a novel environment. Core body temperature (Tb) and motor activity were monitored using telemetry, animals were killed at different time points, brains and blood removed, and primary cell cultures of the anterior pituitary lobe (AL) were investigated. Analyses were performed using immunohistochemistry, RT-PCR, and cytokine-specific bioassays.

**Results:**

Stress stimulation by a novel environment increased NF-IL6-immunoreactivity (IR) in the pituitary’s perivascular macrophages and hypothalamic paraventricular cells and a rise in Tb lasting approximately 2 h. LPS stimulation lead to NF-IL6-IR in several additional cell types including ACTH-IR-positive corticotrope cells *in vivo* and *in vitro*. Two other proinflammatory transcription factors, namely signal transducer and activator of transcription (STAT)3 and NFκB, were significantly activated and partially colocalized with NF-IL6-IR in cells of the AL only after LPS-stimulation, but not following psychological stress. *In vitro* NF-IL6-activation was associated with induction and secretion of TNFα in folliculostellate cells, which could be antagonized by the JAK-STAT-inhibitor AG490.

**Conclusions:**

We revealed, for the first time, that NF-IL6 activation occurs not only during inflammatory LPS stimulation, but also during psychological stress, that is, a novel environment. Both stressors were associated with time-dependent activation of NF-IL6 in different cell types of the brain and the pituitary. Moreover, while NF-IL6-IR was partially linked to STAT3 and NFκB activation, TNFα production, and ACTH-IR after LPS stimulation; this was not the case after exposure to a novel environment, suggesting distinct underlying signaling pathways. Overall, NF-IL6 can be used as a broad activation marker in the brain and might be of interest for therapeutic approaches not only during inflammatory but also psychological stress.

## Background

Inflammatory transcription factors are commonly used as important brain cell activation markers during infection and inflammation to investigate immune-to-brain communication [1-7] and represent promising targets for therapeutic approaches during infectious and inflammatory insults [8-11]. However, information about the physiological role of these transcription factors for the brain during inflammatory and psychological stress is limited. As such, previous studies revealed important implications for pivotal inflammatory transcription factors including nuclear factor (NF)κB and signal transducer and activator of transcription (STAT)3 in fever inducing pathways [8,10]. In concert with endogenous pyrogens, belonging largely to the cytokine family, exogenous pathogen-associated molecular patterns derived from viruses and bacteria [2,3,12-14] lead to a characteristic transcription factor-mediated activation pattern in the brain [5,6,15], and brain inflammation. Among others, this response is linked to the induction of prostaglandin-dependent fever through several autonomic pathways, activating effector organs [16,17].

Recently, we observed that another inflammatory transcription factor, namely NF-interleukin (IL)6, was induced in brain structures implicated in the febrile response but also in HPA (hypothalamic-pituitary-adrenal)-axis activation including the median eminence (ME) and the pituitary, in a time-dependent manner. We hypothesized that it may play a role in the manifestation or even termination of fever as well as HPA-axis activity and brain inflammation [1]. This response was accompanied by the hypothalamic expression of important brain inflammatory target genes including the rate limiting enzymes in prostaglandin synthesis, for example, cyclooxygenase 2 and microsomal prostaglandin synthase.

In addition to inflammatory stimuli, such as LPS [1,18-20] or viral infections [2,21], brain NF-IL6-expression and activation/nuclear translocation were previously found to be increased in neurons during dehydration [22], in astrocytes, microglia, and neurons after kainic acid-trauma [23], via potassium chloride-induced activity of neurons [24], as neuronal response to axonal injury [25] or by neurotransmitter-induced activation of astrocytes [26]. Recently, we have also shown that brain stab-trauma increases nuclear NF-IL6-IR in the cortex [8]. Interestingly, NF-IL6 has also been implicated in excitotoxic brain injury [23], hypoxia [27], microglia-mediated neurotoxic effects [28], and memory consolidation [29] suggesting a crucial role for this inflammatory transcription factor in the brain, and specifically in brain inflammation.

Whether activation of inflammatory transcription factors also occurs during psychological stress has been previously investigated for STAT3 and NFκB [30,31] but remains unknown for NF-IL6.

Here, we analyzed, for the first time, the precise spatiotemporal activation of the pivotal inflammatory transcription factor NF-IL6 during exposure to a psychological stressor, that is, a novel environment, in comparison to the inflammatory stimulus lipopolysaccharide (LPS). This study revealed a distinct activation pattern in different cell types, such that only perivascular macrophages were activated by both stimuli, but endothelial cells and corticotropes were exclusively activated by LPS stimulation. Moreover, using a primary cell culture of the anterior pituitary lobe we showed that LPS-induced NF-IL6 and STAT3 activation might be involved in TNFα expression by folliculostellate cells. Overall, our data illustrate the pleiotropic role of NF-IL6 in response to different types of stressors. This has important implications for therapeutic strategies, while NF-IL6-immunohistochemistry will also serve as a useful activation marker for future studies investigating inflammatory processes in the brain.

## Methods

### Animals

Male Wistar rats (*rattus norvegicus* spec.) with a body weight (BW) of 200 ± 50 g or 250 ± 50 g (cell cultures) were used for all experiments. The rats originated from an in-house breeding colony with parental animals obtained from Charles River WIGA (Sulzfeld, Germany). Animal care, breeding, and experimental procedures were conducted according to the guidelines approved by the local Ethics committee (ethics approval number GI 18/2 - 51/2008).

Animals were individually housed for the duration of the experiment in a climate chamber that was controlled for temperature and humidity (Weiss Umwelttechnik GmbH, Typ 10'US/+5 - +40 DU, Germany) at an ambient temperature of 25°C and 50% humidity on an 12:12 h light–dark cycle (lights off at 19:00). Animals had constant access to water and powdered standard lab chow (ssniff Spezialdiäten GmbH Soest, Germany) and were implanted with intra-abdominal radio transmitters for measurement of core body temperature (Tb) and motor activity suitable for rats (T-4000 E-Mitter®/ER-4000 Receiver; Respironics Inc-MiniMitter, Bend, OR, USA). Implantation of transmitters was performed about 8 days before the experiment as previously reported [1], using a cocktail of ketamine hydrochloride (50 mg/kg, Pharmacia Upjohn), medetomidine (5 mg/kg, Pfizer; Albrecht), and acepromazine, (0.5 mg/kg) as anesthetic (i.p.). An automatic data acquisition system was used (VitalView, Respironics Inc-MiniMitter, Bend, OR, USA). Rats were handled extensively for at least 3 days prior to the experiment for habituation.

### Treatment and experimental protocols

Rats were intraperitoneally (i.p.) injected with LPS (100 μg/kg or 1 mg/kg BW; derived from Escherichia coli, serotype 0128:B12, Sigma Chemicals, Deisenhofen, Germany) diluted in sterile pyrogen-free 0.9% PBS (Dulbecco’s Phosphate Buffered Saline, PAA, D-Cölbe) at a total injection volume of 1 mL per animal. Control animals received the equivalent volume of PBS (0.1 M, pH 7.4). All injections were performed between 09:00 and 10:00. At different time points (2, 4, 8, 10, and 24 h p.i.) animals were euthanized by terminal anesthesia with pentobarbital (i.p.; approximately 100 mg/kg, Merial GmbH, Hallbergmoos, Germany) and transcardially perfused with ice-cold 0.9% saline.

For the novel environment-stress experiment, rats were placed into an empty cage (without any food, water, or bedding) or stayed in their original environment as a control as previously described [32]. At different time points (30 (*n* = 3), 60 (*n* = 6), 90 (*n* = 3), 120 (*n* = 3), 240 (*n* = 6) min after stress exposure or controls after 60 (*n* = 6) and 240 (*n* = 6) min), rats were euthanized by terminal anesthesia and perfusion. Blood samples were collected with a sterile heparinized syringe via cardiac puncture under anesthesia. These experiments were performed between 10:00 and 14:00.

After perfusion of all animals, pituitaries and brains were quickly removed, frozen separately in powdered dry ice, and stored at −55°C until analysis.

### Tissue processing

Coronal 20 μm brain and pituitary sections were cut on a cryostat (model HM 500, Microm, Walldorf, Germany) encompassing several hypothalamic brain structures including the subfornical organ (SFO), the hypothalamic paraventricular nucleus (PVN), the median eminence (ME), and the pituitary (anterior lobe (AL), intermediate lobe (IL), posterior lobe (PL)) using the stereotactic rat brain atlas of Paxinos and Watson (1998) as reference [33]. The sections were thaw-mounted on poly-L-lysine-coated glass slides and stored at −55°C until processing.

### Immunohistochemistry

Frozen brain sections were air-dried for 7 min at room temperature (RT), immersion-fixed in 2% paraformaldehyde (Merck, Darmstadt, Germany), diluted in PBS for 10 min, and washed three times in PBS. Thereafter, the sections were incubated for 1 h with a blocking solution, consisting of PBS, containing 10% normal donkey serum (NDS; Biozol, Eching, Germany) and 0.1% triton X-100 (no triton X-100 for goat NF-IL6) at RT. Double IHC was performed for analyses of NF-IL6 immunoreactivity in endothelial cells, astrocytes, activated microglia, perivascular macrophages, and neurons. The primary antibodies (NF-IL6 or TNFα) were mixed with the respective antibody to detect specific cell marker proteins (dilutions in Table 1) and sections were incubated for 20 to 22 h at 4°C. After another three PBS washes, NF-IL6 was visualized with Cy3-conjugated anti-goat or anti-rabbit IgG (cat. 705-165-147 and 711-165-152, respectively; Jackson ImmunoResearch, West Grove, PA, USA). Cell-type markers were detected with Alexa-488-conjugated anti-rabbit, anti-mouse, anti-sheep, or anti-goat IgG (cat. A11055, A21202, A11015, and A11055, respectively; MoBiTec GmbH, Goettingen, Germany) as secondary antibodies (1:500 dilution each). Subsequently, sections were incubated for 10 min with 4.6-diamidino-2-phenylindole (DAPI, 1:1,000 dilution in PBS) to stain cell nuclei (Mobitec GmbH, Göttingen, Germany), visualize surrounding tissue, and also to demonstrate nuclear localization of NF-IL6 IR. Finally, all sections were dipped in a glycerol/PBS solution (Citifluor LTD, London, UK), cover slipped (glass cover slips), and stored (at 4°C) until microscopic analysis. All primary antibodies have been used previously and proved to show specific signals [1,34].

**Table 1:** Dilutions for the primary antibodies used for immunohistochemistry or immunocytochemistry

**Antigen**	**Species, type**	**Dilution**	**Catalog number, manufacturer**
NF-IL6	Goat polyclonal IgG	1:250	cat. sc-150-G; Santa Cruz Biotechnology, Santa Cruz, CA, USA
NF-IL6	Rabbit polyclonal IgG	1:9000	cat. sc-150; Santa Cruz Biotechnology, Santa Cruz, CA, USA
STAT3	Rabbit polyclonal IgG	1:8000	cat. sc-21876; Santa Cruz Biotechnology, Santa Cruz, CA, USA
NFκB	Goat polyclonal IgG	1:500	cat. sc-372; Santa Cruz Biotechnology, Santa Cruz, CA, USA
VWF	Sheep polyclonal IgG	1:3000	cat. SARTW-IG; Affinity Biologicals, Ancaster, Canada
GFAP	Mouse monoclonal IgG	1:2000	cat. MAB3402; Millipore, Billerica, MA, USA
CD68 (ED1)	Mouse monoclonal IgG	1:1000	cat. MCA341R; AbD Serotec, Oxford, United Kingdom
CD163 (ED2)	Mouse monoclonal IgG	1:500	cat. MCA342R; AbD Serotec, Oxford, United Kingdom
NOS1	Rabbit polyclonal IgG	1:500	cat. sc-648; Santa Cruz Biotechnology, Santa Cruz, CA, USA
ACTH (1–24)	Rabbit polyclonal IgG	1:2000	Gift from Dr. Blähser, Institute of Anatomy and Cytobiology, JLU Giessen, Germany (Blähser, 1988)
Anti- S100	Rabbit polyclonal IgG	1:250	cat. S2644 Sigma-Aldrich, Munich, Germany
TNFα	Goat polyclonal IgG	1:2000	cat. AF-510-NA R&D Systems, MN, USA

#### Microscopical analysis

A light/fluorescent Olympus BX50 microscope (Olympus Optical, Hamburg, Germany) was used with a black and white Spot Insight camera (Diagnostic Instruments, Visitron Systems, Puchheim, Germany) to acquire images. Digital rat brain maps were arranged for overviews by the adjustment of corresponding rat brain levels taken from the digital rat brain atlas of Paxinos and Watson (1998). Microphotographs for each set of experiment were taken in series for the relevant time points of stimulated and control sections at the same time using the same exposure time (MetaMorph 5.05 software). Image editing software was applied to combine the individual images into the RGB color figure plates (MetaMorph 5.05), to adjust brightness and contrast for better representation and to store the images as TIFF files (Adobe Photoshop 5.05).

#### Semi-quantitative analysis of nuclear NF-IL6 immunoreactivity

Relative values of nuclear NF-IL6 immunoreactivity are presented as estimates for the density of their labeling. Three (PVN, pituitary), one to three (SFO), or two to four (LPS stimulation, pituitary) sections per animal for three to six animals per group were analyzed (Tables 2 and 3). Evaluation was directly performed for each set of experiment at the same time. A five-point scale was used to rate the data as means (2 to 4 sections) of the means (3 to 6 animals): +++ (5), high density of nuclear signals; ++ (4), moderate density; + (3), low density; ± (2), single nuclear signals in some cases; and - (1) no nuclear signals.

**Table 2:** Semi-quantitative analyses of nuclear NF-IL6 immunoreactivity after novel environment stress (stress)

	**Time (min)**						
**Brain structure**	**Stress**					** Control**	
	**30**	**60**	**90**	**120**	**240**	**60**	**240**
**SFO**	++ (3.7)	+ (2.7)	++ (3.7)	+ (3.0)	+ (3.3)	+ (3.0)	± (2.4)
**PVN**	± (2.3)	+ (3.4)	**++ (4.2)**	**++ (4.3)**	± (2.2)	± (2.2)	± (2.0)
**Pituitary**	+ (3.3)	**++ (4.3)**	**+++ (5.0)**	+ (2.7)	+ (2.6)	+ (3.0)	± (2.3)

Semi-quantitative analysis of the amount of nuclear NF-IL6 signals in the subfornical organ (SFO), in the hypothalamic paraventricular nucleus (PVN), and in the anterior lobe of the pituitary gland (pituitary) of rats 30, 60, 90, 120, and 240 min after stress or 30 and 240 min after a control situation (undisturbed). A five-point scale was used to rate the data: + + + (5), high density of nuclear signals; + + (4), moderate density of nuclear signals; + (3), low density; ± (2), single nuclear signals in some cases; - (1), no nuclear signals.

**Table 3:** Semi-quantitative analysis of LPS-induced nuclear NF-IL6 immunoreactivity

**Time (h)**	**100 μg/kg LPS**	**1 mg/kg LPS**	**Control (saline)**
**Anterior lobe of the pituitary**			
2	+ (3.0)		
4	++ (4.4)		
8	+++ (5.0)	+++ (4.6)	± (2.0)
10	++ (4.4)		
24	± (2.0)	± (2.4)	
**Posterior lobe of the pituitary**			
2	± (2.0)		
4	++ (4.1)		
8	+++ (4.8)	++ (3.9)	± (2.0)
10	++ (4.2)		
24	± (2.0)	± (2.2)	

Semi-quantitative analysis of the number of nuclear NF-IL6 signals in the anterior lobe and the posterior lobe of the rat pituitary 2, 4, 8, 10, and 24 h after injection of 100 ug/kg LPS or 8 and 24 h after the injection of 1 mg/kg LPS compared to controls (8 h after injection of saline). A five-point scale was used to rate the data: + + + (5), high density of nuclear signals; + + (4), moderate density of nuclear signals; + (3), low density; ± (2), single nuclear signals in some cases; - (1), no nuclear signals.

### Primary cell culture of the anterior lobe of the pituitary

For each preparation two to three animals were quickly decapitated with a guillotine and the heads were immersed (<20 s) in ice-cold 0.1 M phosphate-buffered saline (PBS; PAA Laboratories GmbH, Coelbe, Germany), pH 7.4. Each pituitary was immediately removed from the skull under low-germ/almost sterile conditions and placed into an ice-cold Petri dish with oxygenated Earle’s Balanced Salt Solution (EBSS; Invitrogen, Darmstadt, Germany). The anterior lobe of the pituitary was removed and placed into a Petri dish filled with ice-cold, oxygenated Hanks Balanced Salt Solution (HBSS) without Ca^2+^ and Mg^2+^ (Biochrom, Berlin, Germany), but supplemented with 20 mM HEPES (Sigma-Aldrich), pH 7.4. Pituitary fragments were then treated with 2 mg/mL dispase-1 (Roche Diagnostics, Mannheim, Germany) in oxygenated HBSS with 20 mM HEPES, pH 7.4 (90 min at 37.0°C). After, the pituitary fragments were washed once with HBSS containing 1.0 mM EDTA (Sigma-Aldrich) to inactivate the enzyme, then washed three times with complete medium, consisting of Dulbecco’s Modified Eagle Medium (DMEM; Invitrogen, Darmstadt, Germany) supplemented with 10.0% FCS (PAA Laboratories GmbH, Coelbe, Germany), penicillin (100 U/mL), streptomycin (0.1 mg/mL), and 4 mM L-glutamine (Biochrom AG, Berlin, Germany). Finally, tissue was mechanically dissociated in 2.0 mL complete medium by repeated trituration with a 1 mL Eppendorf pipette tip. After cell number determination, cells were diluted to approximately 250,000 cells per mL and plated onto prewarmed, poly-L-lysine (0.1 mg/mL; Biochrom AG, Berlin, Germany)-coated glass coverslips (MAGV GmbH, Rabenau, Germany) forming the bottom of a reusable Flexiperm-micro-12 well (6 mm diameter; Greiner Bio-One GmbH, Solingen, Germany) to ensure sufficient cell density despite limited absolute cell number. Cells were cultured in a humidified atmosphere of 5% CO2 and 95% air at 37.0°C. The medium was exchanged the next day to remove cellular debris. Two days later, the culture medium was exchanged with serum-free culture medium to prevent potentially stimulatory effects by its components and experiments were performed the next day. Cell culture conditions were chosen according to in-house procedures for primary neuro-glial cell cultures of the circumventricular organs [35] and adjusted to protocols for primary pituitary cell cultures, previously utilized by others [36-38]. For each experiment, relative cell density was controlled after immunohistochemical staining (5 predefined areas counted; mean of approximately 500 cells).

#### Treatment protocols

Cells were stimulated with LPS (100 μg/mL) or PBS and incubated for 6 h. According to a preliminary time course experiment (data not shown) and results from other primary cell cultures, 6 h proved to be an ideal time point to detect TNFα immunreactivity and its release (16 independent experiments) into the supernatant [39]. In another set of experiments (3 independent experiments, 2 to 3 wells for each treatment), the JAK-STAT-inhibitor AG490 (N-Benzyl-3,4-dihydroxybenzylidenecyano-acetamide; 1,5 μg/μL, 5 mM; Enzo Life Sciences International Inc., PA, USA) or its diluent (25% Cremophor®EL, Polyoxyethylenglyceroltriricinoleat 35, DAC in PBS; into serum-free medium 1:50; Sigma-Aldrich, Munich, Germany) were used to preincubate cells 30 min before control or LPS stimulation. The cells of the anterior lobe of the pituitary were fixed with 4% freshly prepared paraformaldehyde (Merck) in PBS, pH 7.4, for 15 min at RT and immunocytochemistry was performed as previously reported [39] using the same antibodies and dilutions as indicated in Table 1. Supernatants were used for the determination of TNFα.

#### Quantification of ACTH-immunoreactive cells

To investigate the effects of LPS treatment and AG490 pretreatment on the number of ACTH-IR cells we quantified the number of cells in five equal areas using a conventional ocular counting grid. The selection of the areas was standardized: in the middle and on the four outer edges of the cell coated cover slips. First, all ACTH-positive cells and second the total number of DAPI-positive cell nuclei were counted. Data were then calculated as percentage of ACTH-positive cells out of all cells counted for one experiment. Finally, the mean percentage of ACTH-positive cells out of three independent experiments was calculated and used for blotting the data and for statistical analyses.

### Plasma IL-6 and TNFα measurements

IL-6 and TNFα levels were determined by bioassays. The IL-6 assay is based on a dose-dependent growth stimulation by IL-6 on the B9 hybridoma cell line. For the TNFα-bioassay the cytotoxic effect of TNFα on the mouse fibrosarcoma cell line WEHI 164 subclone 13 was used as previously reported [2]. The detection limit of the assay after adjustment for assays dilutions of samples was 3 international units (IU) for IL-6/mL and 6.0 pg/mL for TNFα. TNFα levels in primary cell culture supernatants of anterior rat pituitaries were measured for three independent experiments with 2 to 3 wells for each treatment and adjusted to the relative cell density in percentage to LPS and cremophor-stimulated samples.

### Data analysis

Abdominal temperatures (at 5-min time intervals) and cumulative motor activity (cumulative over 15 min periods) were compared by a two-way repeated measures analysis of variance (ANOVA) followed by an all pairwise Tukey multiple comparison post-hoc test (SIGMA Stat®; Systat Software, Inc., Point Richmond, CA, USA.). Circulating levels of bioactive IL-6, TNFα, ACTH-positive cells, and cumulative motor activity over 240 min were compared by ANOVA followed by Newman-Keuls multiple comparison post-hoc test (GraphPad Prism 5 software; San Diego, CA, USA). *P* < 0.05 was considered statistically significant. All data are presented as means ± SEM.

## Results

### Psychological stress induces hyperthermia, hyperlocomotion, and increased NF-IL6-IR

**Figure 1:**
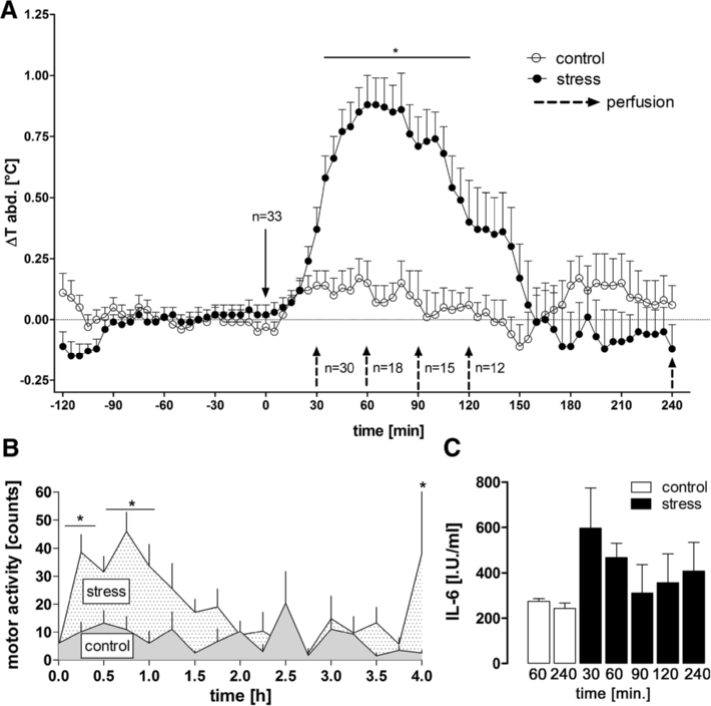
**Tb, motor activity, and plasma IL-6 levels during novel environment stress or control situation in rats. (A)** Novel environment stress (stress) induced a rise in body temperature (Tb). **(B)** This response was preceded and accompanied by increased motor activity (averaged cumulative activity over 15 min for each) but **(C)** no significant rise in plasma interleukin 6 (IL-6)-levels, when compared over time to unstressed control animals. Dashed black arrows indicate changes in the number of animals; this number of animals reduces with time because of animal groups being perfused at the indicated time points. *n* = 3 (30, 90, and 120 min) or 6 (60 and 240 min) for each group of perfusion (stress; control only for 60 and 240 min). For IL-6 *n* = 3 or 6 (60 and 240 min) samples were analyzed *P < 0.05.

Psychological stress induced an increase in Tb from 30 to 120 min (Figure 1A, *P* < 0.05; mean increase 1.03 ± 0.19 C; stress *vs.* control) and an increase in locomotor activity from 30 to 90 min (Figure 1B, *P* < 0.05), as previously reported [32]. Plasma IL-6 levels did not increase over time in the stressed animals (Figure 1C, one way ANOVA).

**Figure 2:**
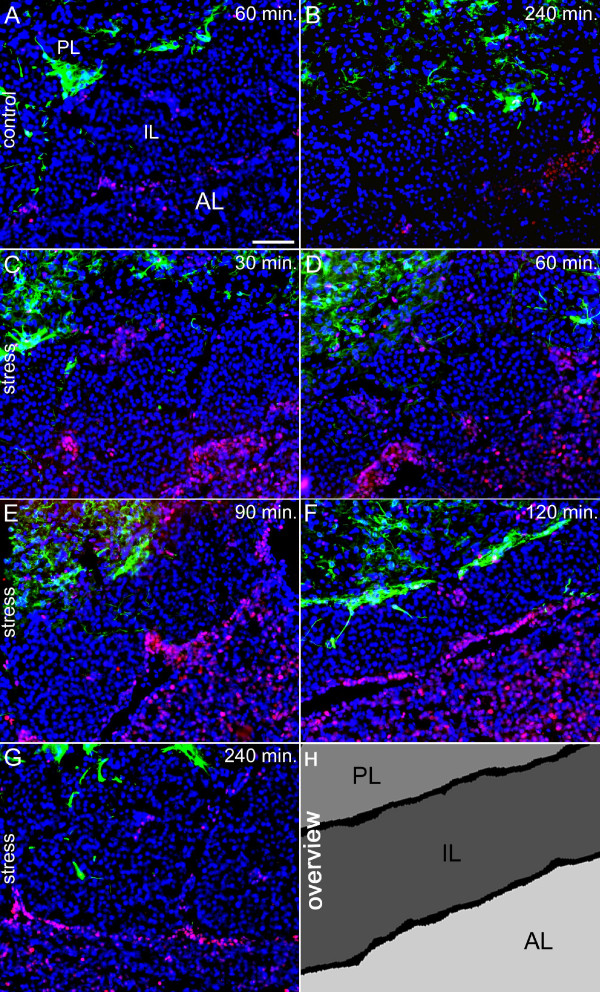
**Novel environment stress (stress) induced a significant increase in nuclear NF-IL6-IR in the pituitary of rats. (A, B)** Some nuclear (DAPI, blue) NF-IL6-IR (red) can be observed in unstimulated control animals. **(C-F)** After novel environment-stress was introduced, nuclear NF-IL6-IR peaked **(D, E)** 60 to 90 min later and **(G)** declined to control levels at 240 min. GFAP detection (green) was used to better visualize the posterior pituitary lobe. **(H)** The schematic overview clearly depicts the substructure of microphotographs, containing all pituitary lobes (anterior lobe (AL), intermediate lobe (IL), and posterior lobe (PL)). Brightness, contrast, and color balance were adjusted for better representation of the actual data. The scale bar in **A** represents 100 μm (applies to **A-G**).

**Figure 3:**
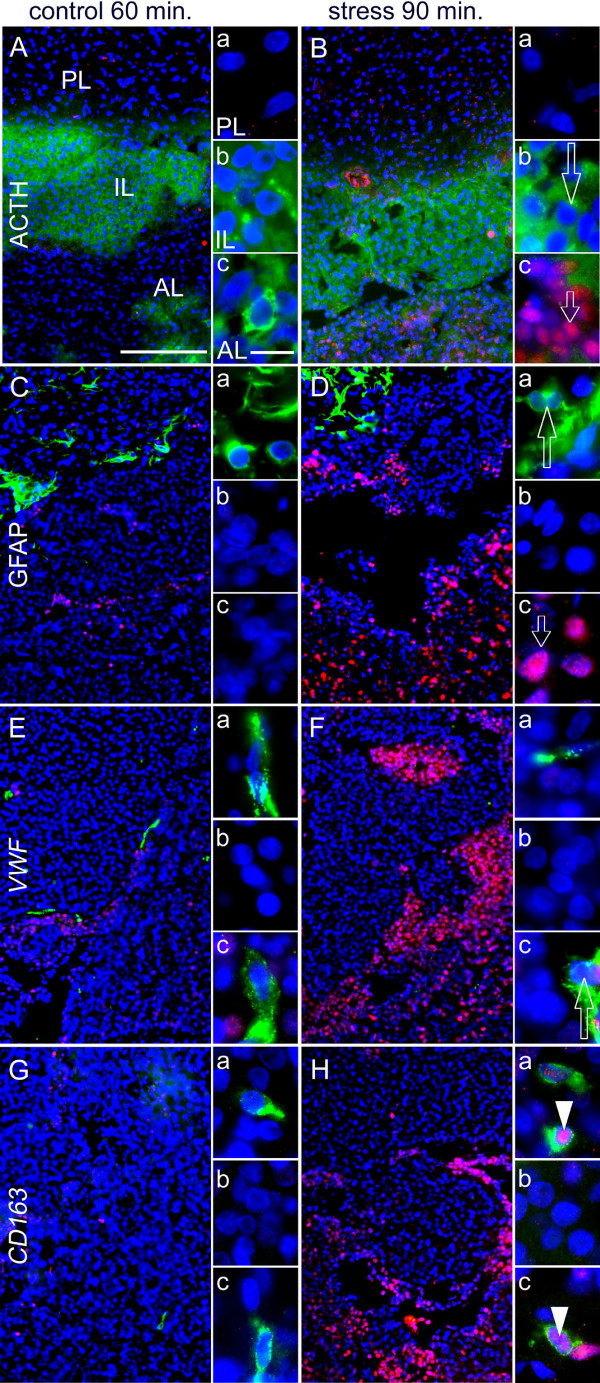
**Novel environment stress (stress) induced nuclear NF-IL6-IR that co-localizes with CD163-positive perivascular macrophages in the rat pituitary.** NF-IL6-IR (red) was co-localized with specific cell marker proteins (green) after novel environment-stress (90 min) or in unstimulated control animals (60 min). **(A-H)** Stress-induced nuclear NF-IL6-IR was not co-localized in corticotrope cells (ACTH; **A**, **B**), astrocytes (GFAP; **C, D**), endothelial cells (VWF; **E, F**) but in perivascular macrophages (CD163; **H**) of the anterior (AL, **c**) and posterior pituitary lobe (PL, **a**). Insets **(a-c)** show high magnifications of the PL, IL, and AL, respectively. Open arrows represent either NF-IL6-IR-negative cells of identified phenotype or some NF-IL6-IR cells of unidentified phenotype (green). White arrow tips show nuclear NF-IL6-IR in perivascular macrophages. Cell nuclei were labeled with DAPI (blue). Brightness, contrast, and color balance were adjusted for better representation of the actual data. The scale bar in **A** 100 μm (applies to **A-H**) and 10 μm for all insets **(a-c)**.

These effects were accompanied by increased novel environment stress-induced nuclear NF-IL6-IR that peaked between 90 and 120 min in the PVN (Additional file 1E,F) and between 60 and 90 min in the pituitary (Figure 2D,E) but was unchanged in other brain structures such as the SFO (Table 2). NF-IL6-IR appeared to be particularly strong lining the intermediate lobe and in the anterior pituitary lobe compared to untreated controls (Figure 2A-G). A semi-quantitative five-point scale evaluation of two sections per rat for three or six (for 60 and 240 min) animals per group confirmed these qualitative observations (Table 2). In this stress paradigm no NF-IL6-positive corticotropes (Figure 3A,B), pituicytes (Figure 3C,D, GFAP), or endothelial cells (Figure 3E,F, VWF) could be observed. Nonetheless, some NF-IL6-IR positive cells co-localized with CD163-stained perivascular macrophages in both the anterior and the posterior pituitary lobe in stressed (Figure 3H) but not control animals (Figure 3G). Neither increased activation of STAT3 nor NFκB could be observed in brains and pituitaries of these animals (data not shown).

### LPS induced NF-IL6 IR in pituitary corticotropes and other cell phenotypes

**Figure 4:**
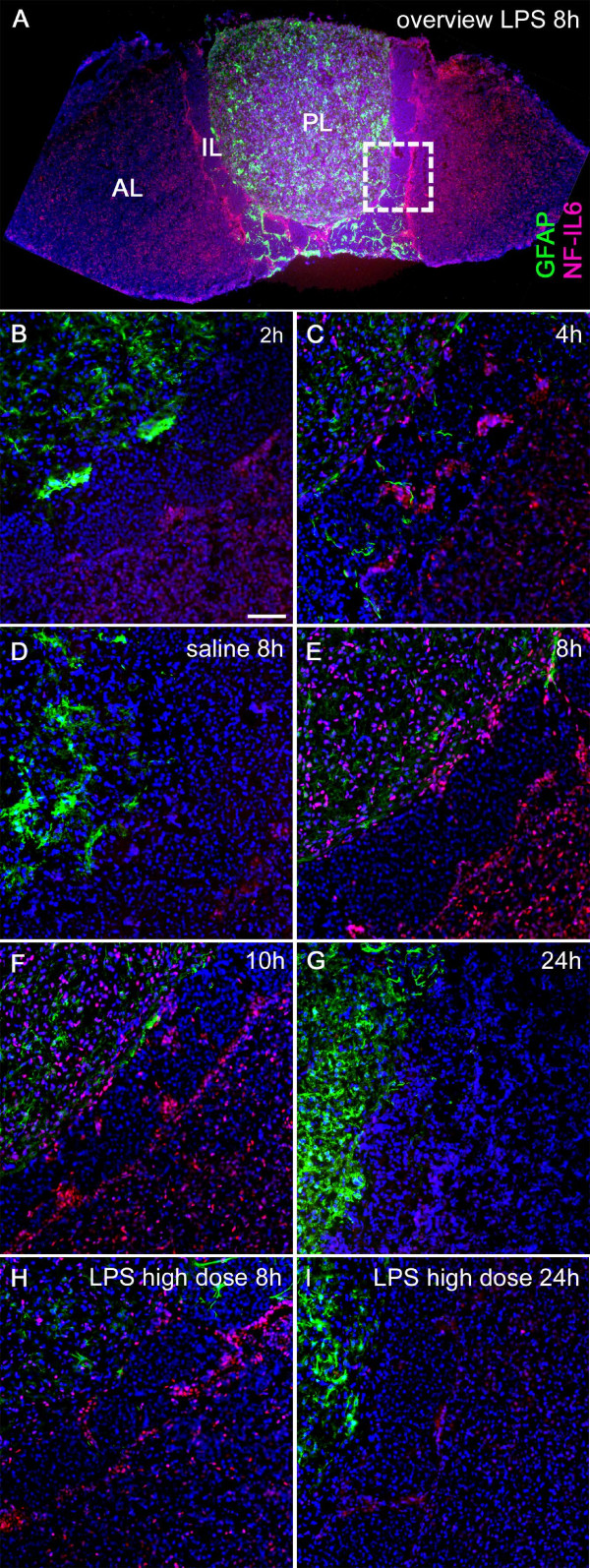
**Time course of LPS-induced NF-IL6-IR (red) in the rat pituitary. (A)** An overview shows typical NF-IL6-IR distribution 8 h after LPS stimulation in the anterior (AL), intermediate (IL), and posterior pituitary lobe (PL). The dashed square indicates respective localization of the following microphotographs at higher magnification. **(B-G)** LPS stimulation (100 μg/kg i.p.) induced NF-IL6-IR, which peaked 8 h after injection **(E)**. Some NF-IL6-IR was also observed 8 h after saline injection **(D)**, which was similar to levels 24 h after LPS stimulation **(G)**. **(H, I)** When injecting a 10× higher LPS dose (1 mg/kg i.p.), the amount of NF-IL6-IR was not further increased at respective time points (8 and 24 h) compared to sections of animals that were stimulated with 100 μg/kg LPS i.p. **(D, E)**. Please note overall strong NF-IL6-IR lining the IL. Cell nuclei were labeled with DAPI (blue) and pituicytes with GFAP (green) for better visualization of the surrounding tissue. Brightness, contrast, and color balance were adjusted for better representation of the actual data. Scale bar in **B** represents 100 μm and applies to **B-I**.

**Figure 5:**
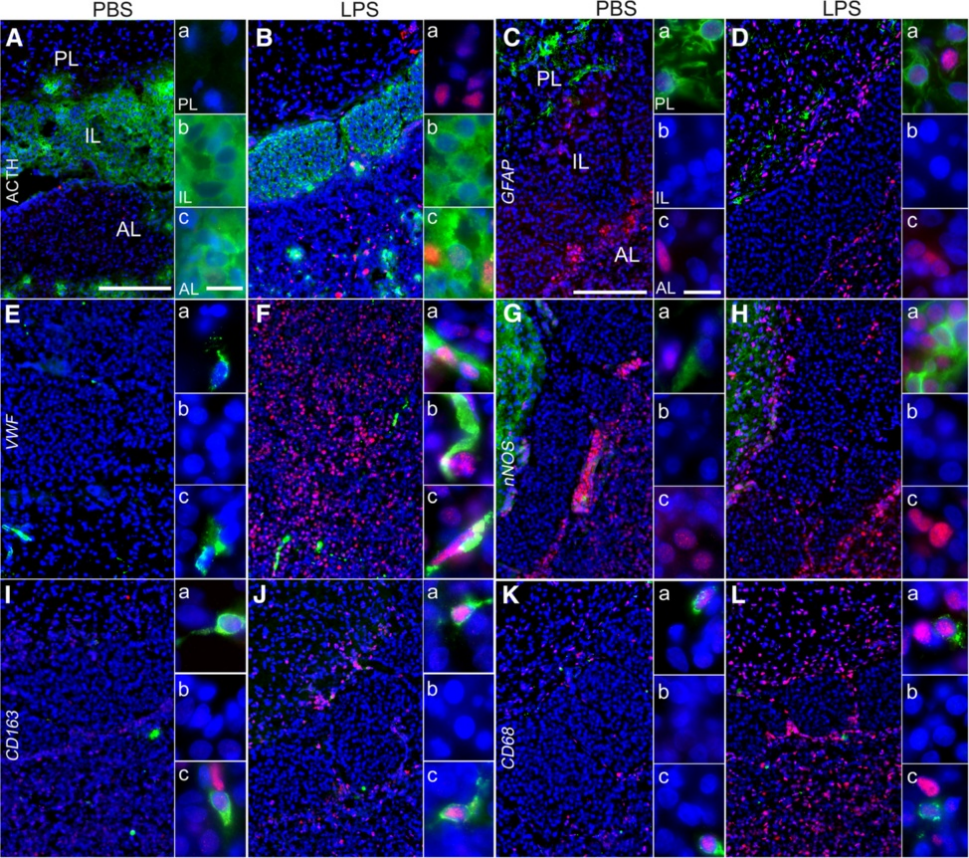
**Cellular phenotypes showing lipopolysaccharide (LPS)-induced NF-IL6-IR in the rat pituitary 8 h after stimulation.** NF-IL6-IR (red) was co-localized with specific cell marker proteins (green) after LPS (100 μg/kg i.p.) or PBS stimulation. **(A-L)** LPS-induced NF-IL6-IR occurred in corticotrope cells (ACTH, **B**), pituicytes (GFAP, **D**), endothelial cells (VWF, **F**), neuronal nitric oxide synthase-expressing neurons (nNOS, **H**), perivascular macrophages (CD163, **J**), and activated macrophages (CD68, **L**) in the rat pituitary. PBS-treated controls also exhibited NF-IL6-IR in pituicytes **(C)**, nNOS-expressing neurons **(G)**, perivascular macrophages **(I)**, and activated macrophages **(K)** to a certain extent. Please note that representative microphotographs depict only very few NF-IL6-IR cells in the intermediate lobe (IL), including endothelial cells **(b)**. Insets **(a-c)** show high magnifications of the PL, IL, and AL, respectively. Cell nuclei were labeled with DAPI (blue). Brightness, contrast, and color balance were adjusted for better representation of the actual data. The scale bars in **A** and **C** represent 100 μm (applies to **A-L**) and 10 μm in all insets (applies to **a-c**). AL, anterior lobe; IL, intermediate lobe; PL, posterior lobe.

Systemic LPS stimulation (100 μg/kg) induced increased NF-IL6-IR in the posterior and anterior pituitary lobe of rats (Figure 4A-C and E-G) compared to saline injected controls (Figure 4D). This IR started to increase at 4 h (Figure 4C) and peaked in its intensity and cell density 8 h after the LPS challenge (Figure 4A and E) followed by a decline after the 10 h (Figure 4F,G) to basal NF-IL6-IR (Table 3). Interestingly, the magnitude of this response was not altered, at 8 and 24 h, when a 10× higher dose of LPS was injected (1 mg/kg, Figure 4H,I). While the intermediate pituitary lobe did not show any NF-IL6-IR, particular strong NF-IL6-IR was observed lining its junctions with both the anterior and the posterior lobe, as can be observed in the overview and higher magnifications of Figures 4 and 5. Semi-quantitative five-point scale evaluation of two to four sections per rat for three animals per group confirmed these qualitative observations (Table 3). Figure 5 shows co-localization of several cell marker proteins with NF-IL6-IR in pituitary sections of LPS- compared to saline-stimulated animals 8 h after injection. We revealed some ACTH-IR positive corticotropes (Figure 5Bc) in the anterior and GFAP-IR positive pituicytes (Figure 5Ca and Da) in the posterior lobe co-localized with nuclear NF-IL6-IR. Endothelial cells (von Willebrand factor, VWF-positive cells) showed NF-IL6-IR-positive nuclei in all parts of the pituitary (Figure 5Fa-c), whereas nNOS positive cells co-localized with NF-IL6-IR only in the posterior lobe (Figure 5Ha). Moreover, CD163 or CD68 positive cells, indicative of the appearance of perivascular macrophages or activated macrophages, respectively, showed nuclear NF-IL6-IR in the anterior and posterior lobe (Figure 5I-L, a and c for each).

### LPS-induced nuclear NF-IL6-IR partly co-localizes with STAT3 or NFκB-IR in the posterior pituitary lobe

**Figure 6:**
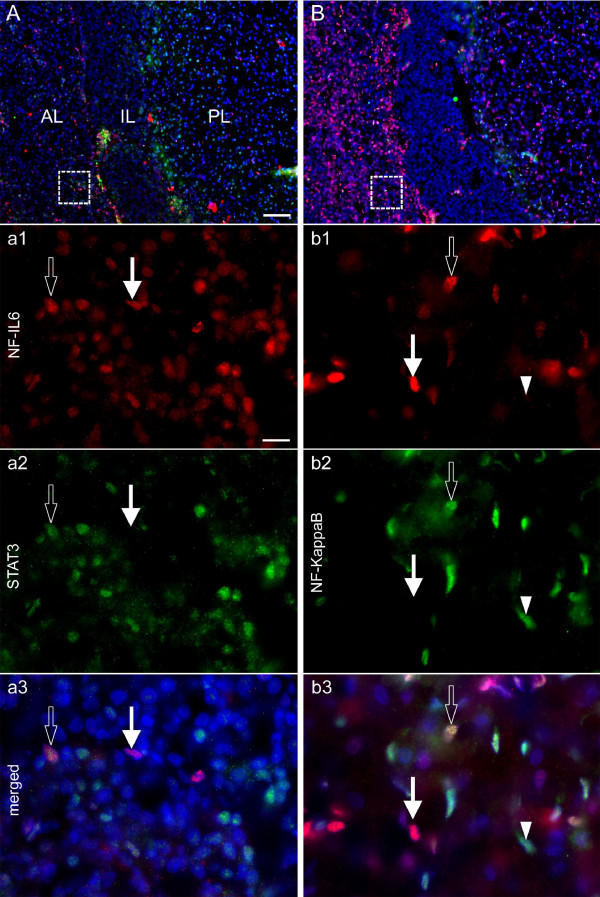
**Co-localization of nuclear NF-IL6-IR with NFκB- or STAT3-IR 2 or 4 h after LPS-stimulation. (A, B)** The overviews depict the neuroanatomical structure and distribution pattern of IR in the anterior lobe (AL), the intermediate lobe (IL), and the posterior lobe (PL) of the rat pituitary. Dashed squares indicate the localization of high magnification **(a, b)**. LPS (100 μg/kg i.p.) induced nuclear (DAPI, blue) NF-IL6-IR (red), which shows some co-localization with both STAT3- and NFκB-IR (both green) as indicated by open arrows in single channels **(a**_**1-2**_**-b**_**1-2**_**)** and the merged overlay **(a**_**3**_**-b**_**3**_**)** 2 (NFκB) or 4 h (STAT3) after stimulation, respectively. Moreover, nuclear NF-IL6-IR can also be seen without co-localization (white arrows) and some single nuclear NFκB-IR occurred with only very weak co-localization with NF-IL6-IR (white arrow tips). Brightness, contrast, and color balance were adjusted for better representation of the actual data. Scale bar in A represents 100 μm (applies to **A**, **B**), 10 μm in a_1_ and applies to **a**_**1-3**_ and **b**_**1-3**_.

Inflammatory transcription factors are known to interact and influence transcriptional activity among each other [40]. Thus, NF-IL6-IR (Figure 6Aa1-Bb1) was co-localized with STAT3 (Figure 6A) and NFκB-IR (Figure 6B) in the pituitary of rats 2 h (NFκB, Figure 6b2-b3) or 4 h (STAT3, Figure 6a2-a3) after LPS stimulation. As previously reported, nuclear STAT3-IR and NFκB-IR were detected all over the pituitary gland excluding the intermediate lobe for NFκB [41,42]. Indeed, NF-IL6-IR co-localized with both transcription factors in the anterior lobe of the pituitary. While some cells only showed NF-IL6-IR, but no STAT3-IR, we did not detect cells that only showed STAT3-IR without NF-IL6-IR (Figure 6a3). As for NFκB-IR, co-localization was detected with either strong or very weak NF-IL6-IR (Figure 6b3). Overall, some NF-IL6-IR in pituitary cells was clearly detected in co-localization with STAT3/NFκB but also without such a co-localization. Novel environment stress did not significantly induce STAT3 or NFκB-IR in the brain (data not shown), although this had previously been reported to be the case after foot shock for STAT3 [31] or restraint stress for NFκB [30]. This most likely was due to the much more moderate stress response to a novel environment in the present study.

### LPS-induced NF-IL6-IR in corticotropes is linked to TNFα induction and release from folliculostellate cells mediated by JAK-STAT3 activation

**Figure 7:**
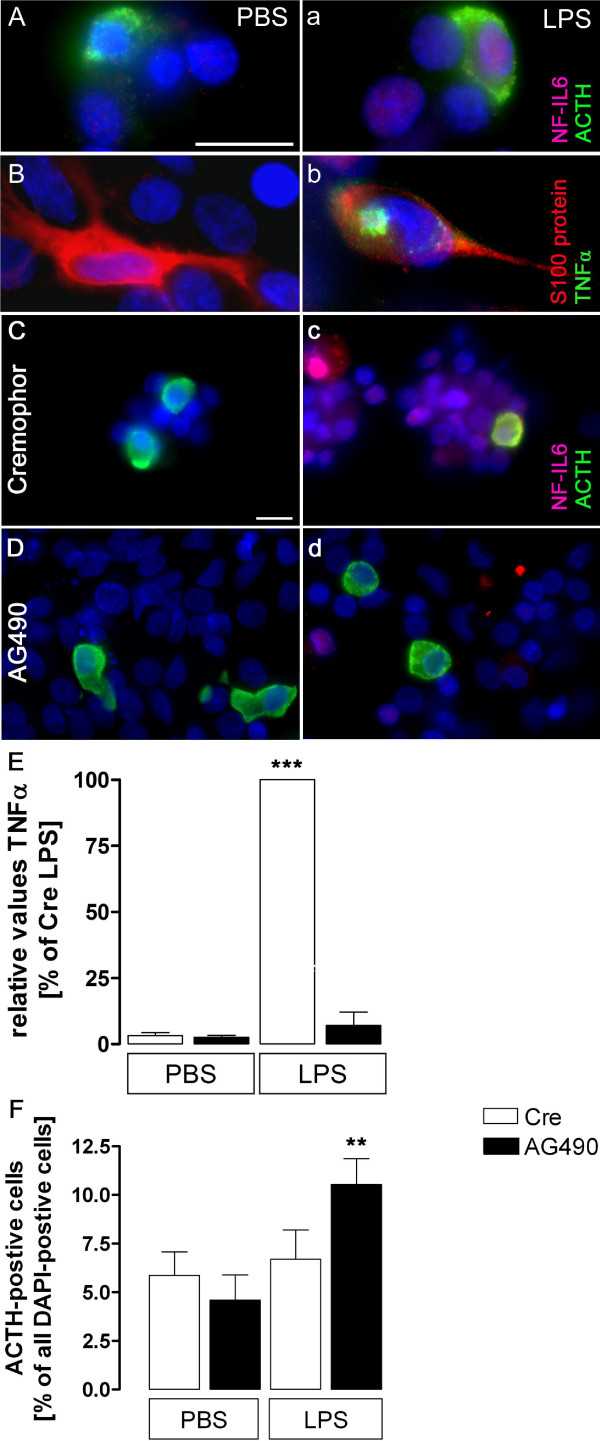
**LPS-induced nuclear NF-IL6-IR is linked to ACTH and increased TNFα in the rat pituitary. (A, a)** Representative microphotographs show that nuclear (DAPI, blue) NF-IL6-IR (red) co-localized with some ACTH-positive cells (green) 6 h after LPS (100 μg/mL, **(a)** but not PBS treatment **(A)** in primary cell cultures of the anterior pituitary lobe. **(B, b)** Moreover, LPS (100 μg/mL) -induced TNFα-IR (green) in S100 protein-positive folliculostellate cells (red) 6 h after stimulation **(b)**, compared to PBS controls **(B)**. **(C, c and D, d)** In a different set of experiments, pretreatment (30 min, 100 μM) with the JAK-STAT inhibitor AG490 reduced LPS (100 μg/mL) -induced NF-IL6-IR partly in ACTH-IR cells (green) of the anterior pituitary lobe 6 h after stimulation **(d)**, compared to solvent (cremophor, Cre) -treated controls **(c)**. Brightness, contrast, and color balance were adjusted for better representation of the actual data. Scale bars in **A** (applies to **A, a, B** and **b**) and **C** (applies to **C, c, D** and **d**) represent 10 μm. **(E)** Relative TNFα concentration in cell culture supernatants, in PBS- or LPS-stimulated and cremophor (Cre) or AG490 preincubated primary cell cultures are presented as percentage of LPS- and Cre-stimulated wells (100%) as calculated for each experiment. Means of two to three wells of the means (of each independent experiment, *n* = 3) were calculated and revealed a dramatic LPS- and Cre-induced TNFα-increase that was abolished by AG490 preincubation. **(F)** Counting of ACTH-IR cells as percentage of all cells in anterior pituitary cell cultures showed a significant increase of ACTH-positive cells after AG490 pretreatment in LPS-stimulated cultures (means of three independent experiments). AG490 did not show any significant effect on the amount of ACTH-positive cells in PBS-treated controls. * LPS & Cre *vs*. LPS & AG490; *** *P* <0.001, ** *P* <0.01.

LPS-induced NF-IL6-IR in corticotrope cells of the anterior pituitary lobe was confirmed 6 h after LPS stimulation of primary pituitary cell cultures (Figure 7a, representative microphotographs out of 16 independent experiments) compared to PBS controls (Figure 7A). This activation was linked to LPS-increased TNFα-IR in S100-postive folliculostellate cells (Figure 7b) and its release into the supernatant of the primary cell cultures (Figure 7E). Pretreatment with the JAK-STAT inhibitor AG490 (100 μM, 30 min before) drastically reduced LPS-induced NF-IL6-IR (Figure 7c,d) and TNFα secretion into the supernatants (Figure 7E), suggesting a role for this signaling pathway for NF-IL6-activation and TNFα production and/or release (*F*_*3, 24*_ = 593.3, *P* < 0.001; AG490 LPS *vs*. Crem LPS; Crem PBS *vs*. Crem LPS; one-way ANOVA followed by Newman-Keuls multiple comparison test). Moreover, AG490 treatment in LPS-stimulated pituitary cells significantly increased the percentage of ACTH-positive cells compared to solvent pretreated (Crem LPS) and control cultures (Crem PBS, AG490 PBS) (Figure 7F; *F*_*3, 24*_ = 9.309; *P* < 0.001, AG490 LPS *vs*. AG490 PBS; *P* < 0.01, AG490 LPS *vs*. Crem PBS, AG490 LPS *vs*. Crem LPS; one-way ANOVA followed by Newman-Keuls multiple comparison test).

## Discussion

In the current study we revealed for the first time that the inflammatory transcription factor NF-IL6 is activated not only in the pituitary after *in vivo* or *in vitro* stimulation with the inflammatory stimulus LPS, but also by novel environment stress in the PVN and the anterior and posterior pituitary lobe. Moreover, our present results further broaden the application portfolio for NF-IL6 as an activation marker, useful for a variety of research questions ranging from LPS inflammation to psychological stress models.

Interestingly, spatiotemporal analyses of NF-IL6-IR cellular phenotypes revealed differences between both types of stressors. On one hand, the time course of NF-IL6-IR was different (peak at 8 h for LPS *vs*. approximately 90 min for psychological stress). On the other, while NF-IL6-IR in perivascular macrophages was detected in both psychological and immune stress, LPS stimulation selectively induced NF-IL6 in pituicytes, nNOS-postive cells, endothelial cells, and corticotropes. In addition, LPS-induced NF-IL6-IR partially co-localized with NFκB and STAT3, and was linked to LPS-induced TNFα-IR in folliculostellate cells of the anterior pituitary lobe and its release in primary pituitary rat cell cultures. In contrast, the novel environment-induced NF-IL6 was not linked to the appearance of other inflammatory transcription factors.

A hallmark of immune-to-brain communication during infection, inflammation, and psychological stress is the concomitant activation of the HPA-axis [43]. First, corticotropin-releasing hormone (CRH) from parvocellular neurons of the hypothalamic paraventricular nucleus (PVN) is released at the level of the median eminence (ME). Second, CRH acts via the portal system on corticotrope cells in the anterior lobe of the pituitary that secrete adrenocorticotrope hormone (ACTH). Third, ACTH induces an increase of corticosteroids in the circulation derived from the adrenal cortex. Overall, this response acts as a negative feedback mechanism, primarily toning down the immune response and limiting HPA-axis activation [44].

Previously, the inflammatory transcription factors STAT3 and NFκB have been implicated in HPA-axis activation [30,31,41,42,45,46]. Moreover, STAT3, NFκB, and NF-IL6 have been implicated in proopiomelanocortin (POMC, precursor of ACTH) and CRH-expression [45-48]. Here, we observed NF-IL6 activation in specific cell types that, in part, co-localized with ACTH immunoreactivity (IR) in the pituitary during LPS-induced inflammation, both *in vivo* and *in vitro*. This observation supports the presumption of a contribution of NF-IL6 in POMC/ACTH expression. However, NF-IL6-IR peaked at a much later time point after LPS (pituitary, 6 to 8 h) or novel environment stress (pituitary, 60 to 90 min; PVN, 90 to 120 min) than expected for its potential role in HPA-axis activation. This stress response starts 15 to 30 min after the inflammatory (LPS, 25 μg/kg) [49] or the psychological stimulus [50] and returns to baseline 480 or 120 min after the respective stimulation. Therefore, if NF-IL6 is involved in this response, it may contribute to a late phase of maintained activation in the pituitary or even its termination. Interestingly, NF-IL6-IR did not co-localize with ACTH IR after psychological stress, indicating that the link between NF-IL6 and the stress axis might not directly involve NF-IL6-mediated ACTH expression. The precise mechanisms remain to be investigated in more detail in the future.

With regard to the effects of psychological stress and the increase in Tb, it remains controversial whether it represents a true fever (for example, a change in the thermoregulatory balance point [51] that is accompanied by an active thermoregulatory response) as opposed to a passive increase of Tb, termed hyperthermia. In favor of a fever-like process, some studies revealed a prostaglandin (PG)-dependent rise in Tb [52-54] and increased thermogenesis by brown adipose tissue [55]. However, these reports seem to pertain to anticipatory stress including the open field model (a similar but stronger stressor compared to the novel environment stress used in this study) but not others, which were reported to be independent of PG synthesis [56,57]. Our present results point to some limited contribution of motor activity to the concomitant increase of Tb in rats after exposure to a novel environment, which in this sense might rather represent hyperthermia than fever.

As mentioned before, NF-IL6-IR cell types differed between the two stress stimuli. We and others have previously shown LPS-induced NF-IL6-IR in the rat or mouse brain *in vivo*[1] and *in vitro*[18]. For instance, brain structures with a leaky blood–brain barrier (BBB) collectively called circumventricular organs (CVOs), such as the SFO, showed NF-IL6-IR in endothelial cells, astrocytes, activated microglia, perivascular macrophages, and neurons. Here, we again observed LPS-stimulated, NF-IL6 activation in perivascular macrophages and endothelial cells, suggesting that circulating mediators such as LPS and cytokines including IL-6 and TNFα might contribute to their stimulation in the pituitary. Since the posterior pituitary belongs to the CVOs and the anterior pituitary is devoid of a BBB, such a direct action is probable and supports the longstanding hypothesis concerning immune-to-brain communication pathways via the humoral pathway [58]. The crucial role of perivascular macrophages [59-61] and endothelial cells [5,62,63] in this response, both of which were activated during systemic inflammation, also suggests a direct influence of circulating inflammatory mediators on the pituitary and its activity. However, psychological stress did not induce NF-IL6-IR in endothelial cells and no significant increase was observed in the SFO, a brain structure prone to detect circulating mediators. In addition, we did not observe a significant increase in circulating IL-6 levels in these animals, although previous results by others did [64]. In our experiments, this indicates that these IL-6-levels do not reach a threshold that would elicit a genomic activation in the brain as previously suggested for STAT3 activation by LPS-induced circulating IL-6 [65]. Moreover, the increase in Tb to an open field exposure was not altered in IL-6 deficient mice [66] confirming that NF-IL6 activation in specific cellular phenotypes and by distinct stress stimuli is dependent on different signaling pathways, for example, a humoral and a neuronal one.

In the posterior pituitary lobe we found NF-IL6-IR pituicytes and cells with strong nNOS-IR with high amounts of NF-IL6-IR cells lining the intermediate lobe (IL) after LPS stimulation. Such a strong nNOS-staining in the posterior lobe and lining the IL has been demonstrated before and was linked to indirect regulation of β-endorphin, a POMC-derived peptide, from the intermediate lobe [36]. Thus, NF-IL6 may play a role in this response; it also has been implicated in production of NO [67], which is known to modulate HPA-axis activity [68].

Another interesting cellular phenotype in the anterior pituitary lobe are S100-protein-positive folliculostellate cells, which also were NF-IL6-IR following LPS administration. These cells have been previously shown to produce IL-6 after LPS stimulation [43] involving the NFκB pathway [69]. Gloddek and colleagues (2001) reported that LPS-induced secretion of ACTH is mediated by IL-6, acting in the pituitary in a paracrine manner [70]. Here, we revealed for the first time folliculostellate cells as one of the sources of TNF production in the pituitary. Although TNFα expression has been observed throughout the pituitary [71-73], the cellular phenotype of TNF-expressing cells has not been determined previously. The shape of TNFα staining resembled the one that had been shown earlier for microglia in other primary cell cultures in the so-called trans-golgi apparatus [39]. Moreover, the release of this particular cytokine was inhibited by the JAK-STAT inhibitor AG490, which was accompanied by reduced NF-IL6-IR. Thus, the JAK-STAT3 and NF-IL6 pathway seem to be involved in this response. The functional role, however, of TNFα in the pituitary for HPA-axis activity remains controversial. Both, inhibitory [74] and stimulatory effects [75] in the pituitary have been reported [43] with TNF-binding sites existing in different cellular phenotypes including folliculostellate cells themselves [76]. Again, this indicates a potential ambivalent role for NF-IL6, either stimulating or inhibiting HPA-axis activity and pituitary/brain function, possibly mediated via TNFα. Here, we found an increased percentage of ACTH-positive cells after AG490 pretreatment in LPS-stimulated cell cultures of the anterior pituitary lobe, while the LPS-induced increase of TNFα levels in supernatant was abolished. This observation indicates that TNFα might, indeed, rather be an inhibitory factor for ACTH expression in response to LPS stimulation.

Recently, NFκB, which is also activated by TNFα, was reported to be crucial for the stress response after LPS and open field stress *in vivo*[30]. While LPS-induced NFκB activity increased IL-6-expression in folliculostellate cells, it inhibited ACTH expression in corticotropes. Mehet et al. (2012) proposed that this striking difference in the NFκB response, depending on the implicated cell phenotypes, represents a regulatory machinery that enhances CRH-activated HPA-axis activity at early stages. When CRH reaches high levels, LPS-induced activation in corticotropes might serve as a limiting inhibitory pathway. Opposed to this report, in another study, TNF-induced NFκB activation seemed to directly contribute to POMC expression *in vitro*[45]. Collectively, POMC expression most likely depends on several transcription factors including NFκB, STAT3, and NF-IL6, since their activity and function depends on physical interaction with each other [40,77]. For instance, NF-IL6 may enhance NFκB-associated signaling [78] and STAT3 enhances NF-IL6 activation [79], knowing that all of them can somehow contribute to POMC expression as mentioned before. In this regard, leukemia inhibitory factor (LIF) was shown to be important for STAT3- and NF-IL6-dependent POMC expression [47,80]. Indeed, LIF contributes to LPS-induced ACTH levels *in vivo*[81]. In the present study, we found some co-localization of STAT3 with NF-IL6 and of NFκB with NF-IL6, suggesting that such interactions might play an important role in the complex function of HPA-axis activation, although a time-controlled subsequent activation of the three inflammatory transcription factors (first NFκB, then STAT3, followed by NF-IL6) also seems to take place. However, some inhibitory functions of NF-IL6 when interacting with STAT3/NFκB has also been described previously [82,83].

## Conclusions

Overall, these present results show, for the first time, an activation of the pivotal inflammatory transcription factor, NF-IL6 following a psychological stressor, and reveal new insights in LPS-induced NF-IL6 activation in the pituitary. NF-IL6 function seems to be, in part, linked to STAT3, NFκB, and HPA-axis activation. Moreover, we reveal evidence for distinct pathways in the spatiotemporal NF-IL6 activation after either psychological or inflammatory stress (that is, humoral *vs*. non-humoral). While brain perivascular macrophages are activated in response to both stressors, endothelial cells, pituicytes, CD68 positive cells, and corticotropes show NF-IL6 activation only after LPS stimulation. The role of NF-IL6/STAT3 in LPS-induced inflammation seems to involve modulation of TNFα expression in folliculostellate cells of the pituitary as shown by inhibition of LPS-induced TNFα levels in cell culture supernatants by the JAK-STAT/NF-IL6 inhibitor AG490. In addition, this treatment, that is, TNFα inhibition, was associated with increased numbers of ACTH-positive cells. Subsequently, not only STAT3, but also NF-IL6 activity might potentially be linked to a modulation of stress-axis activity, but this remains to be confirmed in future studies. As inflammatory transcription factors are important brain cell activation markers and therapeutical targets for a variety of endogenous (glucocorticoids) and exogenous (that is, [9]) anti-inflammatory/inhibitory strategies, these observations are of high interest for a broad context of research areas and questions involving inflammatory as well as psychological stress models and brain inflammation.

## Abbreviations

ACTH: Adrenocorticotrope hormone; AL: Pituitary anterior lobe; BBB: Blood–brain barrier; CD: Cluster of differentiation; Crem: Cremophor; CRH: Corticotropin-releasing hormone; CVOs: Circumventricular organs; DMEM: Dulbecco’s modified eagle medium; EBSS: Earle’s balanced salt solution; GFAP: Glial fibrillary acidic protein; HBSS: Hanks balanced salt solution; HPA-axis: Hypothalamic-pituitary-adrenal-axis; IHC: Immunohistochemistry; IR: Immunoreactivity; IL-6: Interleukin 6; IL: Pituitary intermediate lobe; LPS: Lipopolysaccharide; ME: Median eminence; NFκB: Nuclear factor kappa B; NF-IL6: Nuclear factor IL-6; NOS: Nitric oxide synthase; PVN: Hypothalamic paraventricular nucleus; PBS: Phosphate buffered saline; PG: Prostaglandin; PL: Pituitary posterior lobe; POMC: Proopiomelanocortin; RT: Room temperature; STAT3: Signal transducer and activator of transcription 3; SFO: Subfornical organ; Tb: Core body temperature; TNFα: Tumor necrosis factor alpha; VWF: Von Willebrand factor.

## Competing interests

The authors declare that they have no competing interests.

## Authors’ contributions

Conception and design of the experiments was done by RC, RJ, and GR. FF, RC, RJ, and DJ performed collection, analysis, and interpretation of data. RC, RJ, FF, and DJ contributed to drafting the article or revising it critically for important intellectual content. All authors read and approved the final manuscript.

## Supplementary Material

Additional file 1 **Novel environment (stress) -induced significant increase in NF-IL6-IR in the rat hypothalamic paraventricular nucleus (PVN).** Nuclear (DAPI, blue) NF-IL6-IR (red) was co-localized with the specific cell marker protein for endothelial cells namely von Willebrand factor (VWF, green) after LPS (100 μg/kg i.p.) or PBS stimulation. **(A-G)** LPS stimulation induced a peak in nuclear NF-IL6-IR at 90 to 120 min **(E, F)** after stimulation. This response declined at 240 min **(G)** to control levels **(A, B)**. Insets **(a-g)** represent high magnifications in close vicinity to the third ventricle. Please note some constitutive NF-IL6-staining in ependymal cells, which is not significantly enhanced after the stress stimulus. **(H)** The schematic overview clearly depicts the substructure of microphotographs, containing medial parts of the PVN. Brightness, contrast, and color balance were adjusted for better representation of the actual data. Scale bar in **A** represents 100 μm (applies to **A-G**); 3 V, third ventricle. Click here for file
