# Supplementary figures and images for: Activation of the inflammatory transcription factor nuclear factor interleukin-6 during inflammatory and psychological stress in the brain
Source: J Neuroinflammation. 2013 Nov 26;10:140. doi: 10.1186/1742-2094-10-140 (PMC4222273; doi:10.1186/1742-2094-10-140)

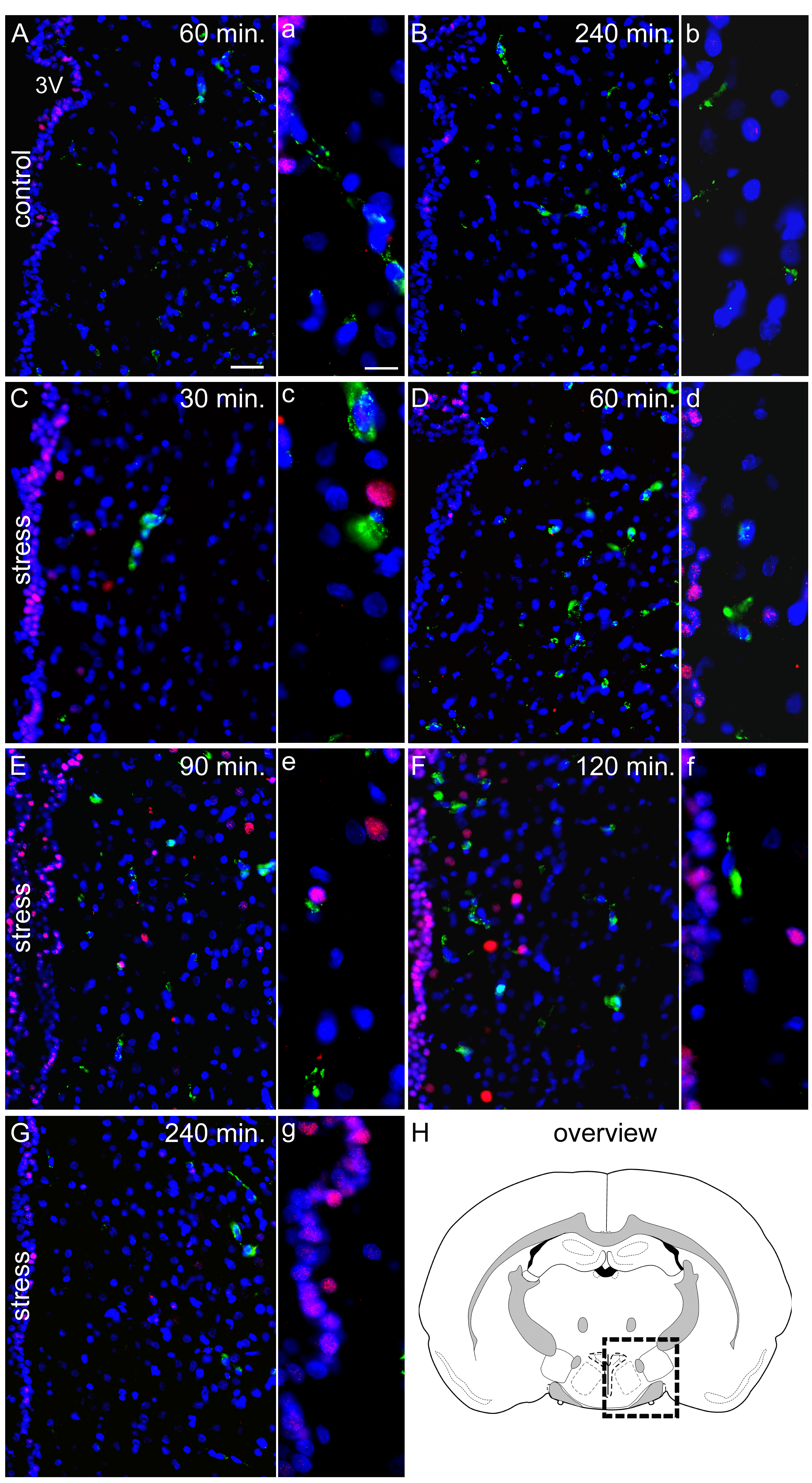

Supplement: Additional file 1 — Novel environment (stress) -induced significant increase in NF-IL6-IR in the rat hypothalamic paraventricular nucleus (PVN). Nuclear (DAPI, blue) NF-IL6-IR (red) was co-localized with the specific cell marker protein for endothelial cells namely von Willebrand factor (VWF, green) after LPS (100 μg/kg i.p.) or PBS stimulation. (A-G) LPS stimulation induced a peak in nuclear NF-IL6-IR at 90 to 120 min (E, F) after stimulation. This response declined at 240 min (G) to control levels (A, B). Insets (a-g) represent high magnifications in close vicinity to the third ventricle. Please note some constitutive NF-IL6-staining in ependymal cells, which is not significantly enhanced after the stress stimulus. (H) The schematic overview clearly depicts the substructure of microphotographs, containing medial parts of the PVN. Brightness, contrast, and color balance were adjusted for better representation of the actual data. Scale bar in A represents 100 μm (applies to A-G); 3 V, third ventricle. [file 1742-2094-10-140-S1.tiff]
